# High incidence of type 1 diabetes, type 2 diabetes and gestational diabetes in Central Iran: A six years results from Semnan health cohort

**DOI:** 10.1016/j.amsu.2022.104749

**Published:** 2022-09-23

**Authors:** Masoudeh Babakhanian, Alireza Razavi, Sajjad Rahimi Pordanjani, Sepehr Hassanabadi, Gholamreza Mohammadi, Abolfazl Fattah

**Affiliations:** a Social Determinants of Health Research Center, Semnan University of Medical Sciences, Semnan, Iran; b Student Research Committee, School of Medicine, Mazandaran University of Medical Sciences, Sari, Iran; c Department of Epidemiology and Biostatistics, School of Medicine, Semnan University of Medical Sciences, Semnan, Iran; d Student Research Committee, School of Nursing and Midwifery, Shahroud University of Medical Sciences, Shahroud, Iran; e Velayat Hospital of Damghan, Semnan University of Medical Sciences, Semnan, Iran

**Keywords:** Incidence, Type 2 diabetes, Type 1 diabetes, Gestational diabetes, Epidemiology

## Abstract

**Introduction:**

Diabetes incidence has increasingly risen in Iran and other low- and middle-income nations in recent decades. In the Semnan Greater Area of Iran, we evaluate the incidence of type 1 diabetes (T1D), type 2 diabetes (T2D), and gestational diabetes during 2015–2020 as well as their six-year trend.

**Methods:**

This is a retrospective analysis of data (n = 820401) from the Integrated Health System (sib) in Semnan province during 2015–2020. All diabetes cases with diagnostic codes based on the International Classification of Diseases 10 (ICD-10) are listed by year. The Grid Search method was used to obtain the exact number and time of points when the incidence of diseases changes significantly (Joinpoints). Average Annual Percent Change-Annual Percentage Change (AAPC-APC) values and slop changes in the estimated regression line with 95% confidence interval were utilized based on diabetes types to determine Joinpoints. P-value < 0.05 is considered statistically significant.

**Results:**

The proportions of diabetes types (T1D, T2D, and gestational diabetes) among 820401 diabetics were 4.18%, 94.84%, and 0.97%, respectively. APC value denotes that T1D has increased by 12.47% per year on average in this period (P-value < 0.01). The incidence of T2D and gestational diabetes has increased between 2015 and 2020 (APC = 15.02 and APC = 136.138, respectively; P-value < 0.1).

**Conclusions:**

In summary, the incidence of diabetes in Semnan province is constantly increasing. T2D, meanwhile, has a higher proportion. Nevertheless, gestational diabetes had the highest increase annually. Well-designed surveys investigating the reasons for diabetes increment especially gestational ones and its burden are needed.

## Highlights

• The incidence of diabetes has been increasing in Iran and other low- and middle-income countries over the past decades.
• The incidence of diabetes in Semnan has been steadily increasing from 2015 through 2020. T2D, meanwhile, has a higher proportion.
• Although the trends in the incidence of T1D, T2D and gestational diabetes were increasing, gestational diabetes increased more than the other types.

## 1 Introduction

Diabetes is a crucial public health issue that is demarcated with insufficient carbohydrate metabolism, protein, and fat due to erratic insulin secretion, insulin resistance secretion, autoimmune response, and pregnancy [1]. Diabetes causes several complications and mortality [2]. As claimed by the latest data from the International Diabetes Federation (IDF) in 2019, nearly 463 million adults in the world live with diabetes [3]. Several estimates point that by 2040, the number of diabetic patients will increase to 642 million worldwide [4]. During the years 1990–2010; the disease's rank has dropped from 15 to 9, which reaches a 92.7% increase in burden over these 20 years [5]. In recent years, diabetes prevalence has increased rapidly due to the aging of society, hereditary background, unhealthy lifestyle, and obesity [6,7]. Diabetes prevalence in individuals over the age of 18 was assessed to be 8.5% in 2014, which has risen significantly over the past three decades, mainly in low- and middle-income nations [4]. In this sense, this trend is more significant in the Middle East and North Africa [8]. A study aimed at estimating the prevalence of diabetes and the number of individuals of all ages with diabetes for the years 2000 and 2030 based on World Health Organization (WHO) data, estimated 20051 diabetic individuals in 2000 in the middle eastern crescent, with a projection of 52794 diabetic patients by 2030 in this region [9]. In the fourth round of the periodic National Survey of Risk Factors for Non-communicable Diseases project in 2011 (SuRFNCD-2011), the prevalence of diabetes in Iran was estimated at 11.4% of the adult population, an increase of 35% compared to the 2005 report [10]. In 2011, about 4.5 million adults in Iran lived with diabetes, so that more than a quarter of this population had just been diagnosed [10]. It is also assessed that by 2030, 9.2 million people in Iran will develop diabetes [11]. Numerous complications caused by diabetes and the continuous and significant increase in the prevalence of diabetes in Iran demonstrate the high burden of this disease [12]. There are reports of the epidemiology of diabetes mellitus (DM) in Iran [2], but data on diabetes prevalence and its types in Iran are limited. Recently, among 30202 patients with diabetes in Iran in 2016, Prospective Analysis from First Nationwide Diabetes Report of the National Program for Prevention and Control of Diabetes (NPPCD-2016) reported a ratio of type 1 diabetes (T1D), type 2 diabetes (T2D), and other types of diabetes to 11.4%, 85.5%, and 1.3%, respectively [12].

Occasionally, epidemiologic research has fallen victim to common pitfalls and neglected some important subjects. Adhering to established guidelines can aid the healthcare system in developing, validating, and deploying precise clinical prediction models for diabetes epidemiology. Studying longitudinal data is vital to determining disease incidence and enabling casual inferences [13]. Semnan province is another region in Iran where diabetes has a significant prevalence. Although some reports evaluated gestational diabetes mellitus (GDM) in this province [14], comprehensive, current, and identifying information on the types of this disease is not available in Semnan. This study aimed to estimate the incidence and six-year trend of diabetes and its types in Semnan province.

## 2 Methods

### 2.1 Study design

This retrospective cohort study was operated on all diabetic patients in Semnan province from 2015 through 2020. Ethics approval was granted by the Ethical Committee (ID: IR.SEMUMS.REC.1398.172) and the Research Council of 10.13039/501100007103Semnan University of Medical Sciences and all methods were accomplished in accordance with the pertinent guidelines and legislations. Data collection was through the Integrated Health System (sib) only and no patients were interviewed. This study is reported in line with the STROCSS criteria [15].

### 2.2 Patients

All diabetic patients (T1D, T2D [DM type 1 and 2] & gestational diabetes) regardless of sex and age living in Semnan province registered in the Integrated Health System (sib) of Semnan University of Medical Sciences (https://sib.semums.ac.ir) or Shahroud University of Medical Sciences (https://sib.shmu.ac.ir) during 2015–2020 were evaluated to enter the study through the census method. DM is defined as A1C ≥ 6.5% or fasting plasma glucose (FPG) ≥ 126 mg/dl or 2-h plasma glucose ≥200 mg/dl or a random plasma glucose ≥200 mg/dl [16]. Also, gestational diabetes is defined as disturbed glucose tolerance first detected in pregnancy by a 75-g oral glucose tolerance test (oGTT) under standardized conditions with quality-confident measurement of glucose from venous plasma [17]. All registered cases older than 18 years with T1D, T2D, and gestational diabetes diagnosis by year with diagnostic code based on International Classification of Diseases 10 (ICD-10) (code E10 [insulin-dependent DM]: only T1D, E11 [non-insulin-dependent DM]: only T2D, and O24 for gestational diabetes [(in childbirth and puerperium) includes pre-existing DM (insulin-dependent), pre-existing DM (non-insulin-dependent), pre-existing malnutrition-related DM, unspecified pre-existing DM, DM arising in pregnancy and unspecified DM in pregnancy]) [18], were received from the health deputies of Semnan and Shahroud Universities of Medical Sciences. Exclusion criteria included other types of insulin-dependent DM (code: E10) [brittle DM, juvenile onset DM, and ketosis-prone DM), other types of non-insulin-dependent DM (code: E11) [maturity onset, nonketotic], malnutrition related DM (code: E12), other specified DM (code: E13) [diabetes NOS], unspecified DM (code: E14), neonatal DM (code: P70.2), glycosuria (NO synthase (NOS): R81 and renal: E74.8), impaired glucose tolerance (code: R73.0), post-surgical hypoinsulinemia (code: E89.1), and lack of access to spatial and temporal information related to diabetes and failure to document patient information in the sib system.

### 2.3 Statistical analysis

The Statistical Package for the Social Sciences (SPSS Inc., version 22.0, Chicago, IL, USA) was used for descriptive analysis. Central and dispersion (mean and standard deviation) indices were used to express quantitative data and frequency (percentage), and charts were used to express qualitative data. Incidence was defined by multiplying the proportion of the population at risk in the same period and region by the number of new cases of the disease in a given period and region by10 ^6^. To analyze the time trend, the incidence rate per 100000 individuals during 2015–2020 for the whole province and separately by type of diabetes (T1D, T2D, gestational diabetes) was calculated, initially. The Grid Search method was used to obtain the exact number and time of points when the incidence of diseases changes significantly (Joinpoints Regression Program version 4.9.0.0). The p-value was calculated using the Monte Carlo method and with 4499 repetitions. Also, Bayesian information criterion (BIC) and Sum of Squared Errors (SEE) were considered to evaluate the precision of the models and select the best model [19]. To identify the exact time of joins, Average Annual Percent Change-Annual Percentage Change (AAPC-APC) values and slop changes in the estimated regression line with 95% confidence interval for the annual disease course during 2015–2020 were obtained by type of diabetes. The threshold for statistical significance was assigned to <0.05.

## 3 Results

In the present study, after applying the inclusion and exclusion criteria, 34315 patients with T1D, 778090 with T2D, and 7996 patients with gestational diabetes were registered in the sib system during 2015–2020. Table 1 shows the number of cases and the incidence of each case by year in Semnan province.

**Table 1:** Frequency distribution and incidence of diabetes in Semnan province during 2015–2020.

Year	Type 1 diabetes		Type 2 diabetes		Gestational diabetes	
	Frequency	Incidence	Frequency	Incidence	Frequency	Incidence
2015	3989	0.61	75203	11.6	93	0.01
2016	4646	0.69	107857	16.2	180	0.02
2017	5404	0.77	129726	18.6	319	0.04
2018	6119	0.92	143344	21.7	1236	0.18
2019	6871	1.02	156817	23.4	2492	0.37
2020	7286	1.06	165143	24.02	3676	0.53

**Fig. 1:**
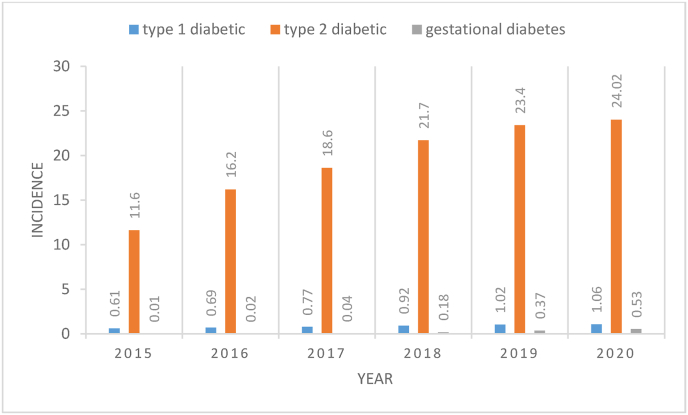
Frequency of diabetes per 100000 people during 2015–2020.

Fig. 1 shows the types of diabetes per 100000 people over six years. As a consideration, each year has the highest incidence of T2D and the lowest incidence of gestational diabetes.

**Fig. 2:**
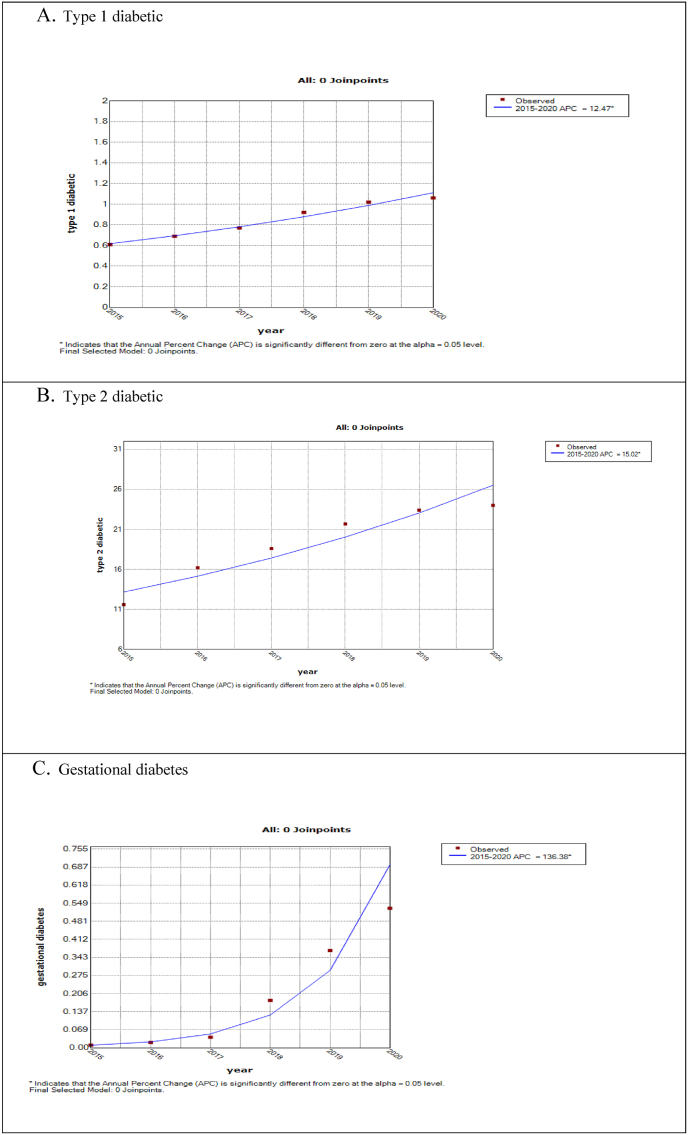
Temporal Analysis with Joinpoint Regression Models Fitted to Crude Incidence Rate for diabetics in Semnan Province (2015–2020).

Table 2 and Fig. 2 show the results related to the time trend of the incidence of diabetes in Semnan province during the years 2015–2020. The incidence of T1D for Semnan province (Fig. 2-A) in the years 2020–2015 has been significantly increased. The estimated APC value in this period was 12.47, which implies the average in this time period has increased by 12.47% per year. The time course of T2D is also shown in Fig. 2-B. The incidence of T2D during 2015–2020 has been associated with a significant increase (APC = 15.02). The time course of gestational diabetes is also shown in Fig. 2-C. The incidence of gestational diabetes during the years 2015–2020 has been associated with a significant increase (APC = 136.138).

**Table 2:** More details of temporal analysis with join-point regression models fitted to incidence of diabetics in Semnan province (2015–2020).

Type of diabetics	Time frame of trend	APC		P-value
		Point estimate	95% CI	
Type 1	2015–2020	12.47^a^	9.7–15.3	<0.001
Type 2	2015–2020	15.02^a^	7.5–23	<0.1
Gestational diabetes	2015–2020	136.138^a^	94.1–187.9	<0.1

a Indicate the APC and AAPC is significantly different from zero at the alpha = 0.05 level.

## 4 Discussion

Our demonstration that the incidence of diabetes increased significantly from 2015 to 2020, with the highest incidence for T2D and the lowest for gestational diabetes. Our analysis goes further to show that the incidence trends of T1D, T2D, and gestational diabetes increased significantly during these 5 years, with gestational diabetes increasing more than the other types. This study achieved that the increase in the incidence of gestational diabetes during these 5 years can be a warning the healthcare system. However, Surveys on the epidemiology of diabetes in Semnan province are very few and details about the prevalence of diabetes in this province are vague.

In a 2005 prospective cohort study performed for global screening of gestational diabetes on 1310 pregnant women referred from private clinics and community health centers to Fatemieh Hospital in Shahroud, the incidence of gestational diabetes was 4.8% [14]. In the current study, the incidence of gestational diabetes increased exponentially from 0.01 to 0.53 over these 6 years. Evidence suggests that in Semnan province, body mass index (BMI) greater than 30 kg/m2 (OR 4.7, 95% CI 2.8–7.9; P < 0.001), family history of diabetes (odds ratio (OR) 4.6, 95% confidence interval [CI] 2.7–7.8; P < 0.001), macrosomic history (OR 9.3, 95% CI 4.6–18.7; P < 0.001) and age over 30 years (OR 3, 95% CI 1.8–5; P < 0.001) are considered as the most notable risk factors for gestational diabetes [14]. Additional Iranian work implies that waist-to-height ratio and waist circumference (WC) have high power in predicting hyperglycemia. After the age-adjusted OR, along with the presence of hyperglycemia, the waist-to-hip ratio (WHR) had the highest OR (OR 7.74; 95% CI 1.71–15.13). The relationship between WHR and hyperglycemia remained significant even after adjusting for BMI, WC, and menopausal status [20]. Consequently, it could deduce the increased incidence of gestational diabetes in Semnan province. Thus, controlling gestational diabetes incidence requires focusing on inherited factors, unhealthy lifestyles, and obesity.

In the National Program for Prevention and Control of Diabetes-2016 (NPPCD-2016) cohort, T1D, T2D, and other kinds of diabetes were identified in 11.4% (11.0%–11.8%), 85.5% (85.1%–85.9%), and 1.3% (1.2%–1.4%), respectively. Patients with gestational DM accounted for 1.8% (1.7%–1.9) [12]. In the current study, T2D was the most common, whereas gestational diabetes was the least common. In other words, these findings emphasize the importance of paying more attention to T2D management.

Co-occurring diabetes with high blood pressure or obesity is a frequent pattern [21]. The prevalence of obesity in diabetic patients in the NPPCD-2016 study in women and men is 52% and 42.4%, respectively [12]. The rate of obesity and overweight in Iran is growing [22]. In a 2016 report, the prevalence of obesity in the Iranian general population was about 20%, which stands higher among women (30%) than men (17%) [23]. However, there is still not sufficient data in this regard. The need for new preventive strategies aimed at reducing the prevalence of diabetes-related metabolic-cardiac risk factors among Iranian adult patients with diabetes is seriously felt.

Many people with diabetes are still undiagnosed in Iran. In a longitudinal study in Yazd, a province located south of Semnan, the proportion of people with DM who were unaware of their disease was 4.8% (95% CI = 4.1–5.5) [2]. In another study in Yazd [24], the prevalence of undiagnosed diabetes was 9.0% in the general population and 18.6% among Zoroastrians [25]. In Kerman province, a southern province, the prevalence of undiagnosed diabetes is approximately 2.7%, but 25% in northern Iran [26,27]. Considering our study, Although the prevalence of diabetes has been steadily increasing from 2015 to 2020, it is not possible to accurately predict the rate of undiagnosed diabetes in Semnan. Diabetes-related health systems information programs, including the Diabetes Research Center and health campaigns and screening programs throughout the province, can help health managers execute practical programs to prevent and control diabetes and diagnose diabetes early.

According to the findings of a meta-analysis, the global incidence of T1D was 15 per 100000 individuals, with a prevalence of 9.5% (95% CI: 0.07 to 0.12), which was statistically significant [28]. According to the most recent published results on the epidemiology of T1D in Iran, out of 30,202 people with diabetes, 11.4% had T1D, which is considerable [12]. These findings suggest a high burden of T1D on human societies. Our findings back this up, with a 12.47% annual increase in T1D.

An analytical - cross sectional study in 2004 in Semnan province on 288 patients with diabetes showed that blood sugar and A1c hemoglobin levels are not regulated in most patients and diabetes control in these patients is insufficient [29]. Considering that our results show the increase in the incidence of diabetes in Semnan as well as the trend of increasing the annual growth of this disease in Semnan, frequent screenings and timely identification of these patients should be a priority for health institutions in this province.

There have been several limitations in current study. To begin with, this reasearch was performed retrospectively. Due to its nature, bias in selection, recall, and misclassification can provide a lower level of evidence. As a result, the proportions of different forms of diabetes, the incidence of different types, and trends in diabetes rates in this study may differ from the general population, and this limitation should be carefully acknowledged when interpreting the study's fundamental findings. The lack of data on the rates of diabetes complications (such as blindness, amputation, stroke, peripheral vascular disease), socio-demographic information, hospitalization records, or mortality, as well as the cross-sectional nature of the correlations, limits this study. To ascertain the true burden of diabetes in Iran, accurate estimates of these illnesses are essential.

## 5 Conclusions

The current study showed the incidence of diabetes in Semnan province is increasing steadily. T2D, meanwhile, has a higher proportion. Nevertheless, gestational diabetes had the highest increase annually.

## Ethical approval

Ethics approval was granted by the Ethical Committee (ID: IR.SEMUMS.REC.1398.172) and the Research Council of 10.13039/501100007103Semnan University of Medical Sciences and all methods were accomplished in accordance with the pertinent guidelines and legislations.

## Sources of funding

This research received no specific grant from any funding agency in the public, commercial, or not-for-profit sectors.

## Author contributions

All authors contributed significantly and in agreement with the content of the article. All authors were involved in project design, data collection, analysis, statistical analysis, data interpretation and writing the manuscript. All authors read and approved the final, submitted version.

## Registration of research studies

1. Name of the registry: Research Council of Semnan University of Medical Sciences, Semnan, Iran
2. Unique Identifying number or registration ID: IR.SEMUMS.REC.1398.172
3. Hyperlink to your specific registration (must be publicly accessible and will be checked): https://ethics.research.ac.ir/PortalProposalList.php?code=IR.SEMUMS.REC.1398.172&title=&name=&stat=&isAll=&GlobalBackPage=https%3A%2F%2Fwww.google.com%2F

## Guarantor

Abolfazl Fattah.

## Consent

In this study, because the data was collected from a database, there was no need to obtain informed consent from the patients.

## Provenance and peer-review

Not commissioned, externally reviewed.

## Declaration of competing interest

The authors declare that they have no conflict of interest.
